# Engineering stress resistance: advances in glycine betaine production for sustainable agriculture

**DOI:** 10.1007/s44297-025-00044-5

**Published:** 2025-02-26

**Authors:** Fei Zhao, Jinyan Luo, Ezzeldin Ibrahim, Lei Chen, Ying Shen, Muhammad Ibrahim, Wadi B. Alonazi, Jianfei Lu, Yuanchan Luo, Hui Wu

**Affiliations:** 1 State Key Laboratory of Bioreactor Engineering, Shanghai Collaborative Innovation Center for Biomanufacturing Technology, School of Biotechnology, East China University of Science and Technology, 130 Meilong Road, Shanghai, 200237 China; 2 Department of Plant Quarantine, Shanghai Extension and Service Center of Agriculture Technology, Shanghai, 201103 China; 3 Department of Vegetable Diseases Research, Agriculture Research Centre, Plant Pathology Research Institute, Giza, 12916 Egypt; 4 Station for the Plant Protection & Quarantine and Control of Agrochemicals of Zhejiang Province, Hangzhou, 310004 China; 5 Health Administration Department, College of Business Administration, King Saud University, Riyadh, Saudi Arabia; 6 MOE Key Laboratory of Bio-Intelligent Manufacturing, School of Bioengineering, Dalian University of Technology, Dalian, China

**Keywords:** Glycine betaine, Plants, Microorganisms, Synthetic pathways, Genetic engineering, Anti-stress

## Abstract

With the rapid development of industry and frequent extreme weather events, sustainable agricultural production is increasingly confronted with various adversities. Improving the resistance of plants and eco-friendly microbial fertilizers to abiotic stresses is essential for sustainable agricultural development. Glycine betaine (GB), a trimethyl derivative of glycine, plays a crucial role as a cellular osmotic pressure regulator, enhancing the resistance of agricultural plants and microorganisms to stress. Betaine is typically obtained through natural extraction or chemical synthesis; however, the former faces seasonal and geographical limitations, whereas the latter is associated with environmental pollution. Additionally, the post-extraction processes for both methods can increase production costs. Biosynthesis via plants or microorganisms represents a more sustainable approach to betaine production, aligning with the principles of green agriculture. Genetically engineered plants incorporating the betaine synthesis pathway can directly increase GB production, improving plant stress resistance and eliminating the need for costly post-extraction processes. Similarly, genetically engineered microorganisms that construct or enhance the GB synthesis pathway can effectively increase GB production and associated resistance, with relatively simple post-extraction processes that further reduce costs and can be produced continuously. However, the GB synthesis pathway in microorganisms has been less studied. This review summarizes metabolic engineering studies of the GB synthesis pathway in plants and microorganisms and proposes strategies to further increase GB production in engineered microorganisms. In this paper, we review the methods of GB production, its protective effects, its synthesis pathways in organisms, and advancements in genetic engineering research. We also discuss the potential for developing genetically engineered plants or microorganisms for GB synthesis technologies, aiming to lay the groundwork for future applications in sustainable agricultural development.

**Graphical Abstract:**

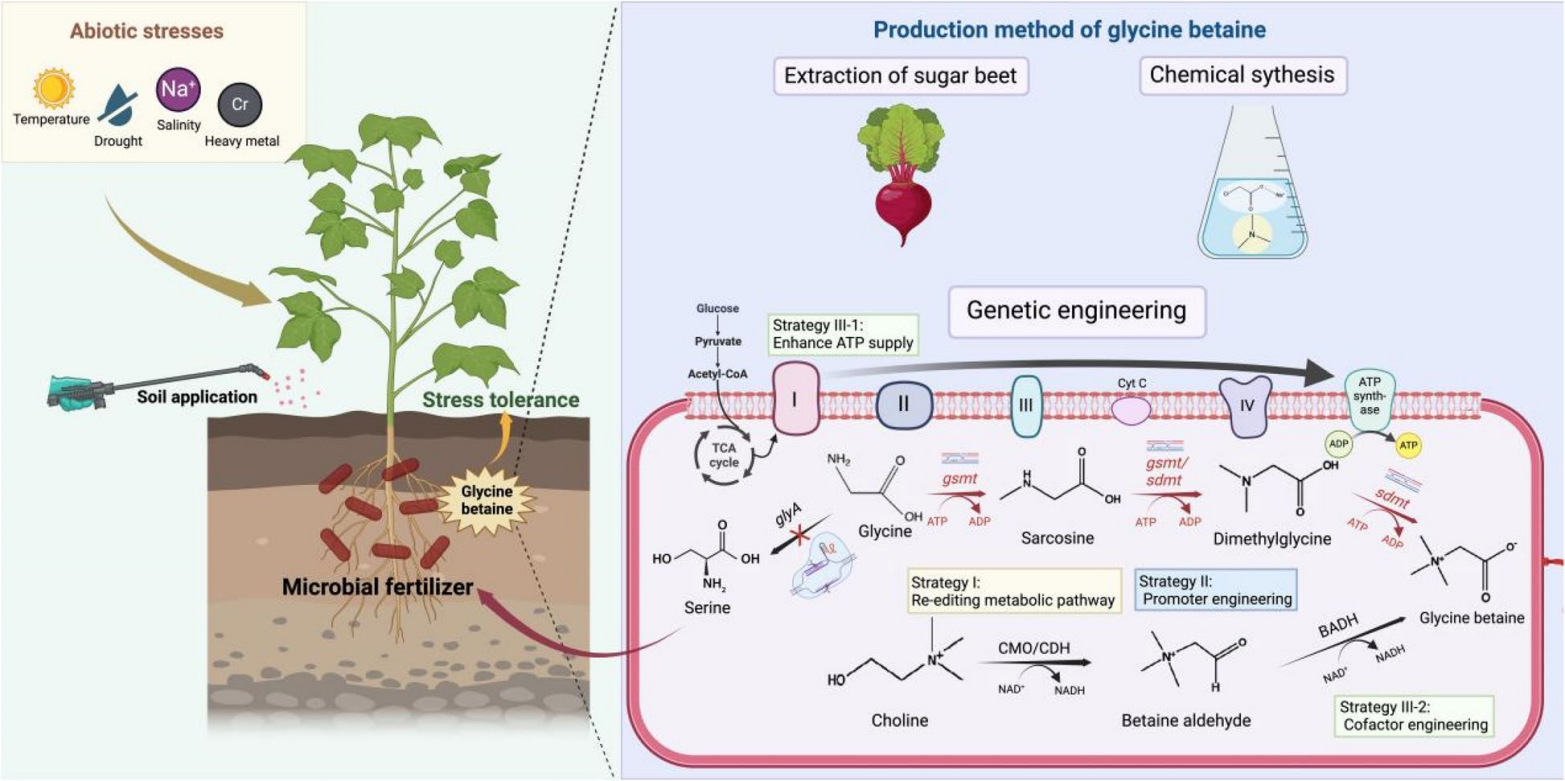

## Introduction

**Fig. 1:**
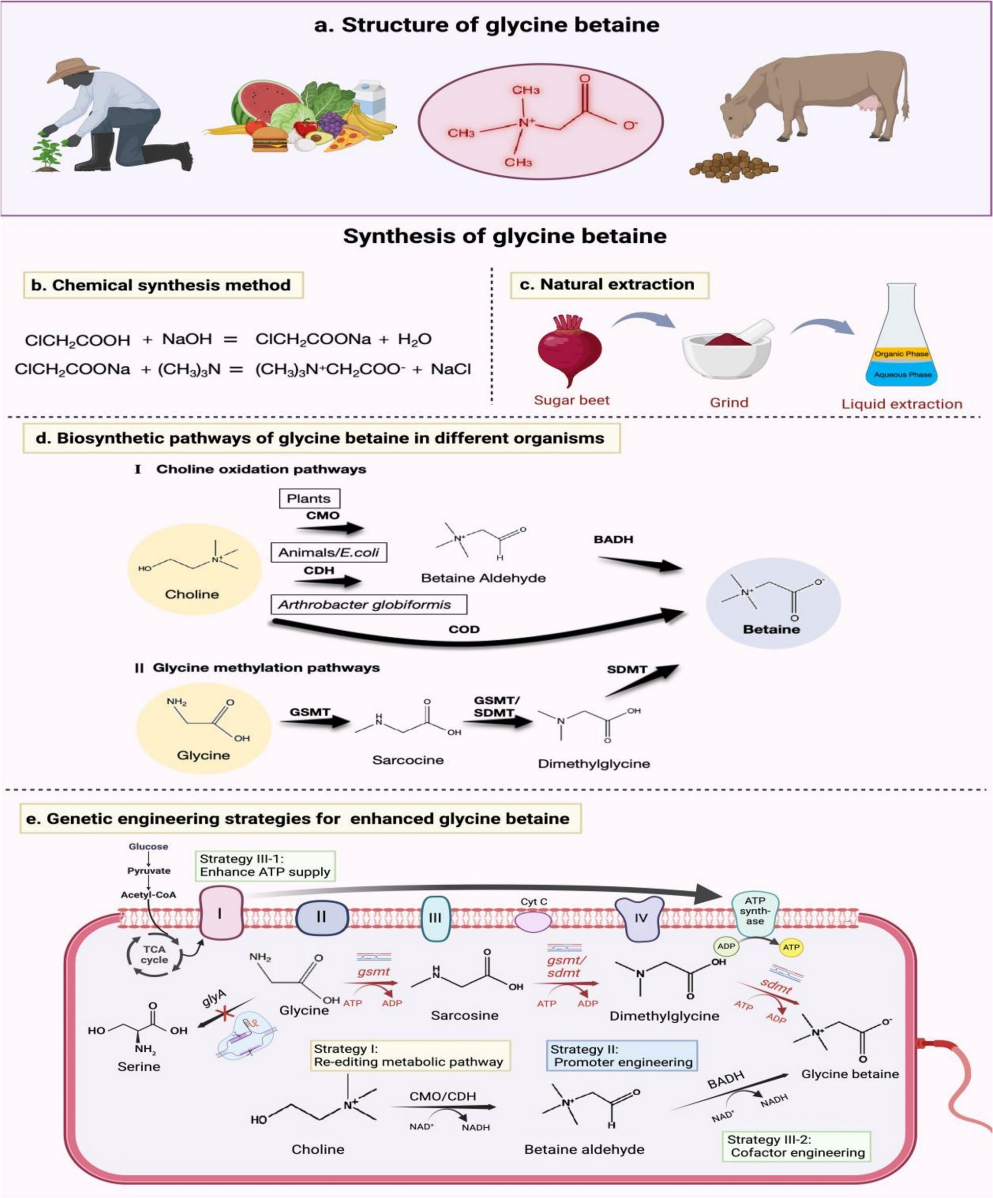
Structure and synthesis of glycine betaine. **a** Structure of glycine betaine; **b** Chemical structure (https://pubchem.ncbi.nlm.nih.gov/); **c** Naturalextraction; **d** Biosynthetic pathways of glycine betaine in different organisms; **e** Genetic engineering strategies for enhancing glycine betaine. CDH: choline dehydrogenase; CMO: choline monooxygenase; BADH: betaine aldehyde; COD: choline oxidase; GSMT: glycine sarcosine methyltransferase; SDMT: sarcosine dimethylglycine methyltransferase. Cyt C: Cytochrome C*; glyA:* glycine hydroxymethyltransferase*.* Figures were created with BioRender.com

In recent years, glycine betaine (GB, Fig. 1a), a trimethyl derivative of glycine, has been widely applied in various industries, including cleaning products, agriculture, food (such as dietary supplements and beverages), and animal feed [1–3]. GB is present in nearly all living organisms and serves as a crucial osmoregulator, methyl donor, and direct nutrient for microorganisms. Owing to the rapid development of industry and frequent extreme weather events, sustainable agricultural production is increasingly facing various adversities (e.g., salinity, cold, drought, and heavy metal pollution). Improving the resistance of plants and eco-friendly microbial fertilizers to abiotic stresses is essential for sustainable agricultural development. GB possesses excellent biocompatibility, a favorable carbon-to-nitrogen ratio, and a high concentration, which can increase the resistance of plants and microorganisms to a wide range of abiotic stresses [4, 5]. GB effectively mitigates the effects of mutagenic compounds and radiation-induced damage, providing protection against abiotic stresses such as salinity, drought, heat, and cold. For example, the exogenous application of GB to the leaves and roots of key crop species (such as rice and potato) has been shown to improve their tolerance to various environmental stresses [6].

With increasing emphasis on sustainable agriculture, microbial fertilizers, recognized for their environmentally friendly properties, are being utilized more widely in agricultural production [7]. As an important bioactive compound, GB plays a unique role in these microbial fertilizers. It enhances the salt tolerance [8], drought tolerance [9], heat resistance [10], and heavy metal resistance [11] of microorganisms that act as active ingredients. This ensures that the active microorganisms in microbial fertilizers can adapt effectively to different abiotic stresses and multiply into a relatively large community. They then maintain their fertilizing properties in complex soil environments, promoting plant growth and increasing plant stress tolerance.

Currently, there are two primary methods for the production of GB in China. The first is chemical synthesis, which involves the reaction of chloroacetic acid and trimethylamine under alkaline conditions. However, this method poses environmental pollution concerns and presents challenges in the later stages of separation and purification [12]. The second method involves natural extraction, primarily from sugar beets; however, this approach is influenced by seasonal and geographical factors, leading to unstable production capacity and insufficient supply [13]. In contrast, plant biosynthesis methods allow the direct utilization of plants for GB production without the need for subsequent extraction steps. Additionally, microbial fermentation methods have garnered significant attention because of their environmental benefits in production [14].

Previous reports have focused on the effects of GB alone on plants [15] or microbes [4] but have not combined the two. Here, we systematically summarize the role of GB in helping microorganisms and plants resist abiotic stresses. Moreover, we suggest that GB-producing microorganisms with resistance properties can be used as raw materials for fertilizers, thus providing a strong impetus for the development of Chinese agriculture and new ideas for research and practice in related fields. In this paper, we review the various methods used to produce GB, its protective effects, its synthesis pathways in plants and microorganisms, and relevant genetic engineering research. We also propose a series of strategies to increase GB production in engineered microorganisms, including the construction of synthetic pathways, promoter engineering, and energy supplementation. Our aim was to gain insights into the efficient and stable enhancement of the protective effects of GB on the sustainable production of agricultural products.

### Role of glycine betaine in stress tolerance in agricultural production

**Fig. 2:**
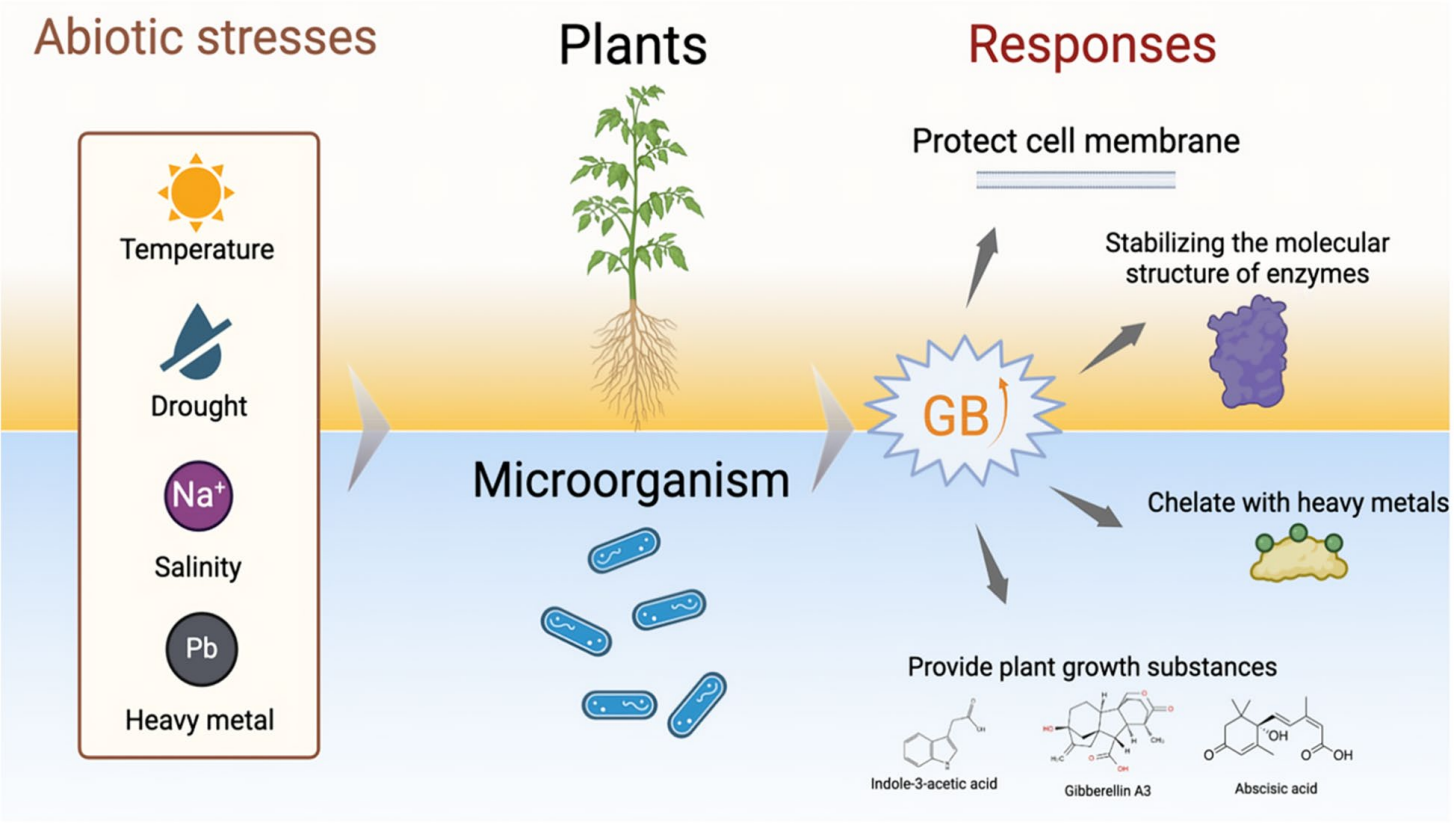
Anti-stress effects of betaine in plants and microorganisms. GB: Glycine betaine. Figures were created by BioRender.com

Numerous studies have shown that GB plays a key role in enhancing the resistance of crops and microorganisms to various stresses, including salinity, drought, heat, and heavy metals, and there are many explanations for the mechanism of GB's anti-stress action, which are mainly related to the following aspects (Fig. 2) [16].

### Improvements in the salt tolerance of crops and microorganisms

The main challenge faced by plants and microorganisms in high-salt environments is the increase in extracellular osmotic pressure, which can lead to intracellular water loss. Under high salt stress, GB can accumulate in plants and microorganisms that naturally synthesize GB [5, 17]. The accumulation of GB helps to regulate the osmotic pressure inside and outside the cell to achieve homeostasis between the internal and external environments, thus preventing cell dehydration [8]. In addition, high-salt environments affect the fluidity and stability of cell membranes. GB is able to interact with phospholipid molecules in the cell membrane, lowering the phase transition temperature of membrane lipids, increasing their stability and reducing salt damage to the membrane [18]. This helps to maintain the cellular functions of material transportation and signaling and ensures the normal metabolic activities of organisms in high-salt environments. Salt stress may also lead to a decrease in enzyme activity in organisms, and GB, as a protective agent, is able to stabilize the molecular structure of enzymes so that they can maintain their activity under high-salt conditions [19] and perform their proper functions. Under 100 mM NaCl salt stress, exogenous GB treatment at 25 mM improved lettuce tolerance to salt stress by increasing total antioxidant and total phenol contents, modulating antioxidant enzyme activities, and altering organic acid and amino acid contents compared with those in plants not treated with GB [20].

### Improvements in the drought tolerance of crops and microorganisms

In arid environments, both plants and microbial cells are subjected to significant osmotic stress and attack from reactive oxygen species (ROS). GB helps organisms sustain normal metabolic functions under these challenging conditions by regulating cellular osmotic pressure [21], protecting the structural integrity of cell membranes, maintaining the activity of essential enzymes [9], and increasing the activity of antioxidant enzymes [22]. When microbial fertilizers containing GB are applied to soil, the microorganisms in the fertilizers can adapt efficiently to adverse soil environments (including arid soil), multiply into a relatively large population and then alter the root morphology and transporter activity of plants to increase the accumulation of nitrate and phosphate, promoting plant growth under drought stress [23]. Additionally, microorganisms produce plant growth-regulating substances (e.g., growth hormones, cytokinin, indoleacetic acid, and ACC deaminase) and antioxidants against drought [24, 86]. Foliar application of GB under water deficit conditions significantly increased both root and shoot weights [25]. Similarly, seedlings derived from GB-treated seeds exhibit greater resistance to drought and are better adapted to arid environments, thereby improving seedling survival rates [26]. In some studies, spraying GB on the leaves of sugarcane seedlings subjected to drought stress alleviated the effects of stress by increasing stomatal conductance, transpiration, photosynthesis, the Fv/Fm (maximum quantum efficiency of photosystem II) ratio, the ΦPSII (effective photochemical quantum yield of photosystem II) and the leaf water potential and mitigated the effects of drought stress on PSII by reducing excess energy and photoinhibition [27].

### Improvements in the heat resistance of crops and microorganisms

The primary challenge that plants and microorganisms face at high temperatures is maintaining the normal function of their biological membranes and proteins. GB helps to preserve the fluidity of cell membranes [10], prevents membrane lipid peroxidation [28], and mitigates protein misfolding at elevated high temperatures by binding to proteins and acting as a molecular chaperone [29]. In addition, it maintains the hydration layer of proteins, protecting their structure and function [30]. GB also plays a role in regulating intracellular energy metabolism pathways, such as ATP synthesis, gluconeogenesis, and fatty acid metabolism, which leads to relatively stable intracellular ATP levels via increased glycogen synthesis and more balanced energy metabolism. This contributes to enhanced heat tolerance in biological systems [31]. Furthermore, during the fermentation process of microbial fertilizers, moderate addition of GB not only improves the fermentation efficiency and yield of microorganisms but also reduces the inhibitory effect of high temperature on microorganisms [24]. In field applications, GB can stabilize the efficacy of microbial fertilizers during the summer and improve the ability of plants to cope with abnormally high temperatures in hot summers [32]. In a three-year field study, Chowdhury et al. [33] reported that split application enhanced the field effectiveness of KNO_3_ and GB foliar spraying compared with a single application with a higher dosage under heat stress. Regardless of the cultivar, the foliar spraying of osmolytes effectively safeguarded the chlorophyll of the flag leaf and sustained a greater level of membrane integrity during the entire post-anthesis stage.

### Improvement in heavy metal resistance in remediated plants and remediated microorganisms

Bioremediation, which combines plants with resistant microorganisms, is an important strategy for addressing heavy metal-contaminated soils. When plants and microorganisms are exposed to heavy metal-contaminated soil, DNA mutations, metal mismatches of proteins, osmotic pressure and reactive oxygen species (ROS) can occur in the cells, resulting in fatal blows to the organisms [34]. GB enhances the uptake and retention of beneficial intracellular ions while reducing the accumulation of heavy metal ions, thereby mitigating their cytotoxic effects. This is achieved by regulating the intracellular osmotic pressure, up-regulating the expression of genes encoding several heavy metal transporter proteins and antioxidant enzymes [34], and forming chelators with heavy metal ions [35].

Research has shown that microbial fertilizers treated with GB significantly increase the survival rate of effective microorganisms in heavy metal-contaminated soils, thereby ensuring the stability of fertilizer efficacy [36]. In addition, this treatment promoted root system development in remediated plants, increased the root length and root surface area, and significantly improved the efficiency of plant uptake of key nutrients such as nitrogen, phosphorus, and potassium [37]. When sugar beet is grown in soil contaminated with Cd (50 mg/kg) and Pb (100 mg/kg), foliar treatment with 1 mM GB can significantly increase the antioxidant content and activity in root and stem tissues and attenuate heavy metal-induced growth inhibition [38]. The application of GB to heavy metal-polluted soil not only improves the diversity index of soil microorganisms but also increases the relative abundance of beneficial microbial populations, thus restoring the ecological function of the soil to a certain extent and contributing to the remediation of heavy metal-polluted soil [39].

In general, GB plays a crucial role in enhancing the effectiveness of microbial fertilizers against different adverse conditions and stabilizing their effectiveness in different application environments. Moreover, GB plays a positive role in the sustainable development of agriculture by directly enhancing the resistance and growth of plants and increasing soil microbial diversity.

### Production method of glycine betaine

#### Chemical synthesis

Chemical synthesis is one of the primary methods currently employed for the production of GB. This process involves converting raw materials, such as sodium hydroxide, trimethylamine and chloroacetic acid, into GB (Fig. 1b). While the GB produced through chemical synthesis is characterized by high purity and the capacity for large-scale production, this method also entails significant cost investments and poses potential environmental pollution risks [13].

#### Natural extraction methods

GB is abundant in many plants, especially sugar beet roots (Fig. 1c), making beet molasses the primary source of GB extraction in China. GB prepared via this method is relatively safe and environmentally friendly compared with chemically synthesized GB; natural GB is favored in the pharmaceutical, cosmetic, and healthcare fields because of its unique advantages [40]. However, this extraction method is susceptible to geographical and seasonal variations and has a relatively complex separation process, resulting in relatively low yields and relatively high production costs.

#### Microbial fermentation

Microbial fermentation is an emerging method for the production of GB that uses specific strains of microorganisms, such as bacteria or yeast, to convert substrates, glycine or choline, to GB through fermentation. For example, in *Escherichia coli*, choline serves as a substrate, which is first converted by choline dehydrogenase (CDH) into betaine aldehyde. This intermediate is then further catalyzed by betaine aldehyde dehydrogenase (BADH) to produce GB (Fig. 1d) [41]. In *Bacillus subtilis,* the initial oxidation step is catalyzed by a different dehydrogenase, specifically a type III alcohol dehydrogenase that is dependent on NAD and utilizes NAD^+^ as an electron acceptor [42].

Subsequent studies have shown that extreme halophiles can convert glycine to GB through a three-step methylation reaction. The first step of the reaction is catalyzed by S-adenosylmethionine (SAM)-dependent glycine sarcosine methyltransferase (GSMT), the second step is catalyzed by either GSMT or SAM-dependent sarcosine dimethylglycine methyltransferase (SDMT), and the third step is catalyzed by SDMT (Fig. 1d) [43]. In addition, these microorganisms can produce plant growth regulators such as indoleacetic acid and gibberellin to promote plant growth and improve plant resistance [44]. They can also degrade residual organophosphorus and other chemical pesticides in the soil and water (e.g., bacteria such as *Xanthobacterium* spp. and *Bacillus* spp., fungi such as *Aspergillus* spp. and *Penicillium* spp.) [45, 46] and immobilize or reduce the toxicity of contaminated heavy metals in the soil [47] for the purpose of environmental remediation. This preparation method overcomes the environmental drawbacks and limitations associated with chemical synthesis and natural extraction, offering a green solution that is not restricted by time or location.

## Biosynthetic pathway of glycine betaine and related genetic engineering

Previous studies have identified choline and glycine as the primary precursors for GB synthesis, which occurs via the choline oxidation pathway and glycine methylation pathway, respectively (Fig. 1d) [48]. With a deeper understanding of the GB synthesis pathway in plants and microorganisms, an increasing number of synthesized genes have been cloned, providing target genes for the establishment of the GB accumulation pathway in plants and microorganisms via genetic engineering. Most likely, because GB was initially developed to enhance its antistress effects on crops, more genetic engineering studies on plant GB synthesis are currently being conducted. On the other hand, owing to their advantages of easy cultivation, short passage time, high productivity, continuous production throughout the year, and convenient post-extraction, microorganisms have attracted increasing attention in recent years for the synthesis of GB via genetically engineered microorganisms. The GB produced by engineered microorganisms can be used in the fields of human health care and cosmetics as well as in agriculture. The current research status of the two synthesis pathways in plants and microorganisms is summarized as follows:

### Choline oxidation

#### Synthesis pathway

In plants, GB is synthesized in two steps: first, choline is converted to betaine aldehyde via a process catalyzed by choline monooxygenase (CMO), and subsequently, betaine aldehyde is converted to GB by betaine aldehyde dehydrogenase (BADH) under aerobic conditions [49]. In higher plants, CMO is found mainly in *Quinoa* and *Amaranthaceae*, and it is hypothesized that different enzyme families that catalyze betaine aldehyde synthesis exist in other higher plants [50]. CMO is the rate-limiting enzyme in the choline oxidation pathway, and its activity directly affects the rate of GB synthesis. The expression of the CMO gene is regulated by a variety of factors, including signals related to stress conditions such as drought and high salinity, as well as phytohormones [51]. The BADH gene, which encodes betaine aldehyde dehydrogenase, has been identified in several species, including spinach [52], barley [53], sugar beet [54] and rice [55]. Notably, BADH is not only involved in GB synthesis but also plays a role in polyamine metabolism because of its functional identity with *ω*-aminoaldehyde dehydrogenase, an enzyme widely found in the polyamine pathway. This functional overlap explains why some plants that do not accumulate GBs, such as wheat and tobacco, still exhibit detectable BADH enzyme activity [56].

In mammals and some microorganisms, such as *E. coli*, there is another choline oxidase system (Fig. 1d) involving choline dehydrogenase (CDH), which parallels the plant synthesis pathway. In this system, CDH first catalyzes the conversion of choline to the intermediate product betaine aldehyde, which is subsequently converted to GB by BADH [57]. In *E. coli*, CDH is encoded by the *betA* gene, whereas BADH is encoded by the *betB* gene. Recently, another choline dehydrogenase, DtCHDH, was also found to catalyze the synthesis of betaine aldehyde in the alga *Dunaliella tertiolecta*, which is the first time that a new choline dehydrogenase has been identified in the phylum Chlorophyceae [58].

In addition, in some specialized microorganisms, GB can be synthesized in a single step. For example, in *Arthrobacter globiformis* [59] and *Arthrobacter pascens* [60], the choline oxidase COD directly catalyzes the oxidative synthesis of GB from choline to GB, producing hydrogen peroxide (H_2_O_2_) as a byproduct. Furthermore, the GB synthesis pathway has also been found in some fungi (e.g., *Aspergillus fumigatus*), where choline is oxidized by the fungus-specific choline oxidases CHOA and BADH to synthesize GB in two steps [61].

#### Genetic engineering

The choline synthesis pathway has been extensively studied, and the genes encoding the enzymes involved in the pathway have been utilized earlier in plant genetic engineering research (Table 1). The earliest work dates back to 1990, when a fragment of the BADH gene was cloned from spinach [62]. Subsequent studies have focused primarily on the modification of COD, CDH, and BADH (Table 1). These modifications can increase the resistance of both dicotyledonous plants, e.g., *Lycopersicon esculentum* [63], *Nicotiana tabacum* [64], *Ipomoea batatas* [65], and *Arabidopsis thaliana* [66], and monocotyledonous plants, e.g., *Oryza sativa* [60], *Zea mays* [67], *Saccharum officinarum* [68], and *Triticum aestivum* [52], by increasing the GB content in these organisms, which can reach 40 mg/g dry weight (DW). In contrast, research on microorganisms has been less extensive, particularly related research focused mainly on the modification of COD and BADH in *E. coli* to help them survive in high-salinity environments and produce GB. For example, Cánovas et al. [69] introduced the *betIBA* gene, which encodes a choline oxidase from *Halomonas elongata* DSM3043, into the defective *E. coli* strain MKH13. This modification enabled the strain to synthesize GB under high-salinity conditions (0.5 mol/L). Additionally, Gu et al. [70] expressed the *gbsAB* gene, encoding a choline oxidase from the halophilic *Halobacillus dabanensis* D-8 T strain, in the *E. coli* PD141 strain. This strain was cultured in saline medium (0.7 mol/L) supplemented with 1 mmol/L choline, resulting in the detection of 10 mM GB.

**Table 1:** Genetic engineering of the choline oxidation pathway in plants and glycine betaine methylation pathway in plants and microorganisms

Specie	Gene	Source of enzyme	Function	References
**Choline oxidation pathway in plants**				
Tomato (*Lycopersicon esculentum*)	*codA* (COD)	*Arthrobacter globiformisde*	Accumulate GB in their leaves and reproductive organs up to 0.3 and 1.2 mmol/g FW, with an average increase in fruit yield of 10–30% during cold stress during cold stress	[90]
Tomato (*L. esculentum*)	*codA* (COD)	*A. globiformisde*	Showed greater resistance to salt stress during seed germination and seedling growth, and higher relative water content, chlorophyll content and proline content of mature leaves	[63]
Tobacco (*Nicotiana tabacum*)	*codA* (COD)	*A. globiformisde*	Betaine as a compound with multifunctional effects attenuates the intensity of lipid peroxidation, even in small quantities, and has a positive effect on tobacco under salt stress conditions on tobacco under salt stress conditions	[64]
Rice (*Oryza sativa*)	*codA* (COD)	*A. globiformisde*	GB's content is 2.12 µmol/g DW, with 50% of the transgenic plants survived under salt stress conditions and had seed production	[91]
Rice (*O. sativa*)	*cox* (COD)	*A. pascens*	The highest level of GB accumulation (up to 2.60 mmol/g DW in the stress-inducible promoter lines grown under saline conditions	[60]
Sugarcane (*Saccharum officinarum*)	*betA* (CDH)	*Escherichia coli*	GB's content is 2.6–4.0 µmol/g FW, improving drought tolerance in sugarcane	[68]
Maize (*Zea mays*)	*betA* (CDH)	*E. coli*	The GB content in the seeds of NT plants was approximately 2.12 µmol/g DW	[67]
Sweet Potato (*Ipomoea batatas*)	BADH	*Spinacia oleracea*	The GB content was 3.04 µmol/g FW, which was more than twofold greater than that of wild type, improving tolerance to various abiotic stresses, including salt, oxidative stress, and low temperature	[65]
Wheat *(Triticum aestivum)*	BADH	*Mountain spanich*	Enhancement of wheat tolerance to salt stress, especially protection of chloroplast-like vesicle membranes	[52]
Arabidopsis *(Arabidopsis thaliana)*	BADH	*Suaeda glauca*	The GB content was 40 mg/g DW, Longer roots, increased glycine betaine accumulation and decreased malondialdehyde	[66]
**Glycine betaine methylation pathway in plants and microorganisms**				
** Plants**				
*Arabidopsis thaliana*	GSMT, SDMT	*Methanohalophilus portucalensis*	Enhance tolerance to drought and salt stress	[75]
*Oryza sativa*	GSMT, SDMT	*Aphanothece halophytica*	Chlorophyll content 2.6 times higher than wild type	[83]
*Gossypium*	GSMT, SDMT	*A. halophytica*	Betaine was obtained at twice the rate of the wild type	[84]
*Anabaena*	GSMT, SDMT	*A. halophytica*	The accumulation levels of GB increased with increase in NaCl concentration reaching to ~ 40 nmol/mg FW at 140 mM NaCl	[82]
*Jatropha curcas*	GSMT, SDMT	*Synechococcus*	GB content was about 0.9 nmol/g fresh weight, improving their drought tolerance	[85]
**Microorganism**				
*Escherichia coli* BL21	GSMT, SDMT	*A. halophytica*	Accumulation levels of betaine in the co-expressing cells were 23 µmol/g DW, which was 3- to 5.5-fold higher than those of control cells	[83]
*E. coli*	GSMT, SDMT	*Ectothiorhodopspira halochloris*	Achieved betaine accumulation and improved salt tolerance	[21]
*E. coli* MKH1	GSMT, SDMT	*M. portucalensis* FDF1T	*E.coli* carrying the synthesized genes can express MpGSMT-SDMT more rapidly under high salt conditions, while enhancing the ability to adapt to the external salt stress	[84]
*Pseudomonas*	GSMT, SDMT	*A. halophytica*	Significantly enhanced protection of bacterial cells against temperature reduction to 15 °C and salinity increase to 400 mM NaCl	[85]

*GB* Glycine betaine, *FW* fresh weight, *DW* dry weight, *COD choline oxidase, CDH choline dehydrogenase*, *BADH betaine aldehyde dehydrogenase*, *NT*: nuclear-transformed, *GSMT glycine sarcosine methyltransferase*, *SDMT sarcosine dimethylglycine methyltransferase*

### Glycine methylation

#### Synthesis pathway

Plants are unable to synthesize GB via the glycine methylation pathway, and only a few microorganisms have been reported to utilize this three-step pathway for GB synthesis (Fig. 1d). In this pathway, glycine is first converted to sarcosine by GSMT. Sarcosine is then converted to dimethylglycine by either GSMT or SDMT, and finally, dimethylglycine is converted to GB. In vitro labeling experiments have demonstrated that S-adenosylmethionine is the methyl donor in this reaction [71]. The methyltransferases GSMT and SDMT, which are required for this pathway, have been identified in several extremophilic bacteria, including halophilic methanogenic archaea such as *Methanohalophilus portucalensis*; photosynthetic sulfur bacteria such as *Ectothiorhodopspira halochloris*; salinophilic actinophilic bacteria such as *Actinopolyspora actinomycetes;* and halophilic photosynthetic bacteria such as *Halorhodospira halochloris.* Additionally, cyanobacteria, including *Aphanothece halophytica* and *Synechococcus* sp. WH8102, also possess these enzymes [4, 43]. The methylation pathway for GB synthesis is rare among microorganisms because the methylation reaction is one of the most energy-intensive processes in nature. The regeneration of an active methyl group in the form of S-adenosylmethionine requires the consumption of 12 ATP equivalents by the cell. Several factors may explain the persistence of this natural pathway: (1) Some betaine-dependent anaerobic microorganisms are unable to carry out oxygen-dependent choline oxidation; for example, *M. portucalensis* is unable to internalize choline for oxidation [72]. (2) Certain betaine-dependent microorganisms, such as the halophilic sulfur bacterium *E. halochloris, lack the genes necessary for choline oxidase* [73]. (3) Some extreme halophilic microorganisms possess both the choline oxidation and glycine methylation pathways; the energy-consuming methylation pathway can be used as a contingency pathway, which may serve as a contingency when the precursor for the oxidation pathway, choline, is limited, as observed in the halophilic actinomycete *A. halophile* [74].

#### Genetic engineering

Since the synthesis of GB via the glycine methylation pathway is an important mechanism for salt tolerance in halophilic microorganisms [75, 76], the key methyltransferases of this pathway, GSMT and SDMT, are usually cloned and transferred into plants or other salt-intolerant microorganisms to construct or increase their GB synthesis capacity to increase resistance to adverse conditions (Table 1).

Several halophilic bacteria and diatoms have been identified in which the glycine methylation synthesis pathway occurs naturally (Table 2) [77, 78] and are excellent natural enzyme source gene pools for GSMT and SDMT. A variety of plants (e.g., *Arabidopsis thaliana* [72]*, Anabaena* [79]*, Oryza sativa* [80]*, Gossypium hirsutum* [81]*, and Jatropha curcas* [82] (Table 1) overexpressing GSMT and SDMT from these bacteria increased the GB content in their cells, which in turn effectively improved their stress tolerance, such as salinity, drought, and cold tolerance (some of the major studies are shown in Table 1).

**Table 2:** Microorganisms that synthesize glycine betaine through the glycine methylation pathway

Species	NCBI RefSeq assembly	[Na^+^] opt./(mol/L)	pH opt	References
**Halophilic bacteria**				
* Actinopolyspora halophila* DSM 43834^ T^	GCF_000371785.1	2.56–3.42	ND	[80]
* Arhodomonas aquaeolei* DSM 8974	GCF_000374645.1	1.00–4.00	ND	
* Ectothiorhodospira haloalkaliphila* ATCC 51935^ T^	GCF_000633935.1	0.85–1.03	8.5–10.0	
* Fodinicurvata fenggangensis* DSM 21160^ T^	GCF_000686045.1	0.85	7.5	
* Halorhodospira halochloris*	GCF_002356555.2	2.40–4.62	8.1–9.1	
* Rubidibacter lacunae* KORDI 51-2^ T^	GCF_000473895.1	0.85	ND	
* Saccharomonospora paurometabolica* YIM 90007^ T^	GCF_000231035.2	1.71	ND	
* Thioalkalivibrio paradoxus* ARh 1^ T^	GCF_000227685.2	0.50–1.00	0.30–4.30	
* Methanohalophilus portucalensis* FDF1^T^	GCF_900177455.1	0.51–2.56	6.5–7.5	[95]
* Desulfonatronospira thiodismutans* ASO3-1^ T^	GCF_000174435.1	1.71	9.0	[96]
* H. halophila* SL1^T^	GCF_000015585.1	1.88–3.76	7.4–7.9	[97]
**Others**				
* Thalassiosira pseudonana CCMP1335*^*T*^	GCF_000149405.2	ND	ND	[81]
* Acetobacterium woodii*	GCF_000247605.1	ND	ND	[98]
* Chromohalobacter salexigens DSM 3043*^*T*^	ND	ND	ND	[99]

*ND *data not shown in the published paper, *opt.* optimatized

In recent years, with the promotion of the use of beneficial microorganisms under adverse conditions and the application of GB in the fields of nutraceuticals and cosmetics, research on the promotion of microbial substances capable of producing GB after the introduction of GSMT and SDMT in non- halophilic microorganisms has gradually increased (Table 1). Waditee et al. [83] transferred the GSMT and SDMT genes obtained by cloning from *A. halophytica* into *E. coli* BL21 and reported that the GB content increased 2 ~ 4.5-fold to 23 µmol/g dry weight after transgenesis. By expressing the gene encoding a methyltransferase from *E. halochloris* in *E. coli*, GB accumulation was successfully achieved, and its salt tolerance improved [21]. The GB-synthesizing gene *Mpgsmt-sdmt* from *M. portucalensis* FDF1T was introduced into *E. coli* MKH13, and *E. coli* carrying the synthesizing gene was able to express MpGSMT-Sdmt more rapidly under high salt conditions while enhancing its ability to adapt to external salt stress [84]. Larissa et al. [85] achieved a high level of salinity tolerance in *E. halochloris* by incorporating salt-tolerant N-methyltransferase-encoding genes from the cyanobacterium *A. halophytica* or the *Limonium hybridum* into *Pseudomonas*, significantly enhancing the protection of bacterial cells against a decrease in temperature to 15 °C and an increase in salinity to 400 mM NaCl.

As described above, the intracellular biosynthesis of GB plays an important role in plant and microbial resistance. The introduction of key genes for GB synthesis into plants can directly increase the amount of GB production in plants and effectively increase their ability to resist abiotic stresses, especially in saline and heavy metal-polluted environments. This method eliminates the need for post-extraction and manual application, greatly reducing production and labor costs. The construction of GB-enriched engineered bacteria can increase the ability of weakly resistant strains to cope with abiotic stress and produce more GB, which can be used as raw material for the anti-stress products in the agricultural and pharmaceutical industries year round after simple extraction.

## Future prospects

Since the “13th Five-Year Plan”, China has implemented the "Two Reduction Policies" (which reduce the use of chemical fertilizers and chemical pesticides) and promoted the development of green and sustainable agriculture. Microbial fertilizers have gradually replaced chemical fertilizers and have been rapidly promoted. Since the active ingredients in microbial fertilizers are living microorganisms, when applied in different soils (farmland is increasingly contaminated by chemical fertilizers, pesticide residues, and heavy metals), improving the resistance of the active microorganisms is the key to maintaining the stability of fertilizer effectiveness. For example, plant growth-promoting rhizobacteria (PGPR) can regulate plant growth and development and improve plant tolerance to abiotic stresses through a variety of mechanisms [86, 87]. If PGPR are engineered to survive better in extreme environments, have high colonization rates, and survive for longer periods, they can interact with plants in a durable mutualistic manner, thus further promoting plant resilience to abiotic stresses. In contrast, few studies have investigated genetically engineered microorganisms for synthesizing GB, and these studies have focused on the model organism *E. coli*. Therefore, research on the synthesis of GB with other microorganisms, especially beneficial agricultural microorganisms such as chassis bacteria, needs more attention and research. At present, research on the use of genetically engineered bacteria or genetically engineered plants for the synthesis of GB is basically at the stage of simple introduction of foreign genes encoding key enzymes.

Given the comparatively low level of GB production in engineered *E. coli*, several strategies have been proposed to improve the production of GB in engineered microorganisms on the basis of the synthesis pathway of GB in microorganisms (Fig. 1d). The main strategies for modifying chassis strains are as follows (Fig. 1e).

Strategy I: Re-editing the synthetic pathway. To better stimulate the activity of key synthase genes in chassis strains under extreme environments, key genes (GSMT, SDMT, CMO/CDH, and BADH) of the GB synthesis pathway exogenously derived from extreme bacteria (e.g., salinophilic bacteria) were introduced into the genome via homologous recombination. Moreover, the competing pathways of the precursor (e.g., glycine) were knocked out via CRISPR. For example, by knocking out *glyA*, a gene that synthesizes serine from glycine, glycine can be maintained at high levels, facilitating the synthesis of GB.

Strategy II: Promoter engineering. Promoter engineering is an effective method for optimizing the expression level of a pathway [88]. The screening of promoter and ribosome binding site sequences from different sources and saturation mutagenesis of promoters to select the most productive and resistant promoters can further optimize the synthetic pathway and promote GB production.

Strategy III: Enhancing energy supply. Synthesis is an energy-consuming process. Therefore, providing enough energy is another effective way to increase yields. Owing to the high ATP consumption (12 ATP) of the glycine methylation synthesis pathway, enhancing the expression of ATP synthase on the electron transport chain (ETC) and balancing the carbon flow between the tricarboxylic acid cycle (TCA) and the glycine synthesis pathway through CRISPi are key methods to further increase GB production. The choline oxidation pathway consumes NAD^+^. Therefore, cofactor engineering, which can provide NAD^+^ from the reaction of NADP^+^ and NADH by over-expression of *pntAB* [89], is a good choice to supplement energy for GB synthesis.

The application of these modified strategies integrated into PGPR (an active gradient in microbial fertilizer) will help them to produce more GB, to have a greater ability to counter abiotic stress, to colonize and reproduce more efficiently in adverse soil environments, and to play a crucial role in helping plants grow under abiotic stress, thus providing a great potential way to increase the development of sustainable agriculture.

## Conclusions

In conclusion, GB, as an excellent compatible solute, plays a crucial role in the adaptation of plants and microorganisms to external abiotic stresses (e.g., drought, high salt, heavy metals, high temperature, etc.), which can effectively facilitate the development of sustainable agriculture. The primary methods of GB production currently include chemical synthesis and natural plant extraction, but these methods have shortcomings such as environmental pollution potential or production time limits. The production of GB via engineered microorganisms or plants is an emerging technology because of its ability to overcome the difficulties associated with traditional GB production. Among these methods, the production of GB by engineered microorganisms has been less reported but has greater potential because of its relatively simple modification method, ease of cultivation, short passaging time, high productivity, and continuous production throughout the year. With respect to the currently comparatively low level of GB production in engineered microorganisms, strategies involving re-editing the synthetic pathway, promoter engineering, and increasing the energy supply are proposed to improve GB production. These strategies, which involve the integration of PGPR with microbial fertilizer, provide new possibilities for the development of sustainable agriculture.

## Data Availability

Not applicable.
